# Editorial

**Published:** 1873-09

**Authors:** 


### Editorial.

### AMERICAN DENTAL ASSOCIATION.

The twelfth annual meeting of the American Dental Association, was held at Put in-Bay, August 5th and 8th, inclusive.

There were about one hundred and twenty-five members and delegates in attendance, and almost the same number of the profession who were merely visitors. Nearly all the officers were present.

The meeting was organized on Tuesday morning at 10 o'clock, Dr. Hunt, President in the chair. The meeting was opened with prayer, by Rev. Mr. Bosley. It might not have been amiss for each day's sessions to have had a similar beginning.

The meeting in many respects was a profitable and interesting one, especially to those who were present, and gave attention to the proceedings. We doubt, however, whether those who went ostensibly to the meeting, but were persistently absent from the sessions, derived any benefit further than that accruing from a reduction of traveling and hotel fares.

How much better it would be, if all who attend these meetings would manifest an interest, and put forth the effort they could for the advancement of the object aimed at. It is certainly a great mistake to hold the meetings of this body, at places of public resort ; better by far be at some point with no outside attractions ; or devote the time allotted to the meeting wholly to it, and afterwards devote as much time as desirable to pleasure and recreation. We trust that hereafter there will be an improvement in this respect. The papers read were very good and instructive ; more care had been given in their preparation than has usually been apparent in the papers presented to this body ; in this respect every year shows progress. The discussions upon the various subjects presented, showed investigation and thought. The profession *does* move, progress *is* being made, the tendency is onward and upward. But we are sometimes impatient, that it goes so slowly compared with what it would, if every one whose interest it is, would but take hold and put forth his best efforts, put his shoulder to the wheel and lift, put his hand to the plow and go forward. There are to be found in all departments of human occupation, those who do not build up, who add nothing to the common stock ; but rather seem to aim to tear down that which others have built. We do not think however that the dental profession has more than a due proportion of this class of persons, and we sometimes think not so many as some others.

Though the American Dental Association has accomplished a great deal, and very much that was anticipated by its founders has been realized; yet the question occurs, could it not have done more? within the last three or four years at least; and is it now in a position to accomplish the greatest good for the profession, and to command the respect of the world as the representative body of the Dental Profession? Does it claim that respect and attention from the profession that it should? Are the majority of the best men in the profession devoting their energies for its support and development? Facts forbid an affirmative answer to these queries. If the American Dental Association is degenerating, why is it?

1st. Because its organization is defective, its laws are very imperfect, and its present membership, or that recently assembled at least, refuse to correct any defects or make any changes whatever, except in some unimportant matters. It was not claimed in the formation of this Association, that its rules and regulations were perfect even for that time, and much less would they be for the present. Occasionally a little change has been made, in its organic law, though usually under stress of great opposition, but for the last year or two, there has been determined opposition to any material change.

About five years ago, a committee was appointed to revise the Constitution; they made their report and it was a good one, and in the main it was adopted. There were some points in it, however, that were susceptible of various interpretations or constructions, out of which many difficulties have arisen; and notwithstanding this, there has been an absolute refusal to make the law more explicit, and correct the difficulty, so that the Association is, at present, wholly without a definite policy in some of its most important features, a determination of which will very much modify the Association for good; and a refusal much longer to do so, will result in its entire demoralization. We trust that another year will put it in a better position. The next meeting will be held in Detroit; a much better place for the interests of the Association, than those at which it has been held for three years past.

The officers for the current year are: *Pres.* Prof. T. L. Buckingham, of Philadelphia, Pa; *Vice Pres.* I. Forbes, St. Louis, Mo; *Recording Secretary*, M. S. Dean, Chicago, Ills; *Corresponding Secretary*, J. Taft, Cincinnati, O; *Treasurer*, W. H. Goddard, Louisville, Ky.

The *Executive Committee* consists of: Drs. Geo. H. Cushing, Chicago, R. G. Thomas and Geo L. Field, Detroit, A. B. Robbins, Denver City, Col., S. B. Palmer, Syracuse, N. Y., L. D. Shepard, Boston, Mass., H. A. Smith, Cincinnati, O., S. H. McCall, Binghamton, N. Y., G. B. McDonnald, Conneautville, Pa.

It is very important that any one upon whom special work devolves, for the next meeting, should take time by the forelock, and make thorough preparation ; and so may the next meeting of this body be the best ever held.

### MICHIGAN STATE DENTAL SOCIETY.

This society holds its annual session, beginning on the second Tuesday of Oct., and will continue two or three days, at Ann Arbor. This society was organized in the beginning of state dental societies, and has gone on with persistantly advancing step from its organization to the present, making some definite progress each year. There is we think, no society in the country that makes more exacting demands of its members, than this one ; and certainly none more scrupulous as to the character and standing of those who seek its membership. This is as it should be. There is nothing that will more certainly destroy the character and efficiency of an association, than the admission to its ranks of uneducated, ungrateful, selfish and designing men, men who care for nor seek anything except their own aggrandizement, and personal interests. The prostitution of associative effort to such purposes, will invariably result disastrously. The requirements for membership in the Michigan society, are more stringent than any other with which we are acquainted, and the result is they have a strong body, composed of the best men in the state. They have labored for three or four years for legislation in behalf of the profession, but as yet without securing the desired object ; but by persistent effort this will come, and we are confident they will not give up till it is attained.

They have now in hand a very important work, which will be brought before this meeting, viz: the organization of a dental college in connection with the Michigan University. The plan is to make available, for this enterprise, much of the teaching that already exists in the medical department, having some slight modifications, to more fully meet the demands of the dental department, and then adding two or three chairs on those subjects that particularly pertain to dentistry as a specialty. Such an arrangement if properly inaugurated and carried out, would ultimate in grand results for the profession, not only in Michigan, but throughout the North-West. In order however to be a success, the dental department should not be an appendage to, nor overshadowed by any other interest; but it should be put side by side with the other departments, and especially that of medicine. And in order that this be carried out fully, the dental students should be required to go through the entire medical course, and stand the same examinations as the medical student, and then his special course in addition. Then, and not till then, will there be anything approximating a thorough professional education; and the best results in this direction, will not be attained, till a good literary and scientific education is made a requisite to entering a medical or dental college. If such a requirement should now be made, it would reduce the classes in these institutions at least three-fourths.

We know of no institution in the United States, that is in a more favorable position to make such a requirement, than Michigan University. And we hope that the Michigan Dental Society, will at least suggest the desirableness of such a regulation, so far as dental students are concerned. It is full time that some progress was being made in this direction, and we shall be rejoiced to see Michigan take the initiative.

It is very desirable that the entire force of the profession in the State should be present at that meeting, and thus manifest an interest in an enterprise that would necessarily exert a great and permanent influence for good. Then let every member be on hand, resolved to do what he can for the best interests of his profession.

### MEDICATION MADE EASY.

Our attention has recently been directed to a new method of preparing medicines, whereby the administration is greatly simplified. It consists in properly compounding, dividing and preparing medicines in a vehic e, so that a patient can readily take them. The vehicle is mainly gelatine ; and all medicines whether solid or fluid, that do not readily volatilize, can be prepared in this manner. But we will give the language of the inventor. He says :

"The divided medicines are prepared with the *most scrupulous accuracy*, according to the rules of *Pharmacy*, in conformity with the latest improvements and discoveries in chemistry, and in such a manner that the several squares must of necessity contain the exact quantity of the medicine in question and NO MORE.

The divided medicines being prepared in a solution, every drop necessarily containing an equal amount of the medicine, it is obvious, that after being infused into mathematicaly accurate squares, each square in the solidified mass, must contain accurately measured quantities of the medicine. But not only *soluble* but also *insoluble* medicaments, we are able by this process to reduce in most accurate division into the form of *Divided Medicines.*

This method of preparing DIVIDED MEDICINES enables us to produce squares, each containing 1 grain, $\frac{1}{2}$, $\frac{1}{4}$, $\frac{1}{8}$, $\frac{1}{30}$, $\frac{1}{100}$ and even $\frac{1}{1000}$ part of a grain, and, if so ordered, *even less quantities.*

The great advantages which the divided medicines present are :

1st, They are pure and reliable in weight.

2nd, They are accurately prepared and divided.

3rd, They are from 50 to 100 per cent cheaper than medicines compounded by any other process.

These divided medicines secure to the *physician andpatient* a most important desideratum, being in such form and bulk that a full supply can be carried in the physician's pocket-book. He has his remedies always at hand, and can administer to his patients the exact dose required, without waiting to weigh and compound the medicine, or without waiting until a messenger can go to an apothecary for them."

Now it is quite apparent that a system such as this, simplifies the administration of medicines very much indeed. Something of this kind would certainly be of great benefit to the dentist. A very great educational defect in the dental profession, is an absence of knowledge of remedies, their nature and uses.

The time is near at hand when the dentist will give far more attention to general hygiene, and medication than hitherto, and anything that will tend to bring about such a result, should be received, encouraged and properly used.

### RUBBER! RUBBER!! RUBBER!!!

About once a day, upon an average, queries upon the rubber subject are presented: about the same routine of questions are asked. "Is there no relief from this rubber monopoly, and the extortion that attaches to it?" YES, don't use it,—or use something better,—or if you must use it, use it and fight; but if your courage has too far oozed out for that, then either quit dentistry or meekly go and submit you neck to the yoke, and pay whatever is demanded, and *shut up*, and keep quiet, or you will have your "title clear" revoked. Many poor souls when they have got a license to vulcanize rubber think it is about as good as a passport to kingdom come, and that Josiah is a precious good fellow that he did not take their shirt and breeches when he took their coat.

Many in the profession do not use rubber for artificial dentures and still find something to do. Very many of the best men in the profession do not use it. The most successful men in the profession do not use rubber; the most scientific men do not use it; the best educated men in the profession do not use it. The men in the profession who best understand the proper treatment of the natural teeth, do not use it, especially when there are natural teeth in the mouth. But then the *quacks all* use it. The men who have been made dentists in six weeks all use it. The dentists who don't believe in natural teeth, all use it. The dentists who think natural teeth a nuisance, all use it. The dentists who know nothing about preserving the natural teeth, use it.

All the dentists who advertise to do many things that no body *can do*, use it.

The dentists who advertise to make teeth cheaper and better than any body else, use it.

The dentists who advertise to fill teeth for a dollar and *upwards*, all use it.

The dentists who stuff teeth with amalgam, nearly all use it. And there are some others who use it ! ! Use something better ? Yes. Well, what ? Well, almost anything, the Japanese use hard wood for dental plates, and they stand for several years. Well, really, he who asks such a question, betrays so much ignorance of the resources of the profession that it is a hopeless task to undertake to inform him, and he ought to be placed back in the alphabet of professional edu cation. But then to the man who will use it, and fight if need be, we say go in, but don't fight till the enemy appears ; but when he does, then apply to Dr. S. S. White, who can supply you with one round of ammunition, at least, in the shape of an "*Answer*," which is already prepared, which you should secure and have filed in the court in which prosecution may be commenced. Now this is brief and simple, but it is enough and to the point, and will secure the purpose if followed.

### AMERICAN DENTAL CONVENTION.

The American Dental Convention held its annual session on the 12th, 13th and 14th of August, at Saratoga Springs.

The attendance was not large, but the meeting was an interesting and profitable one. Quite a number of papers was read, discussion upon which was interesting and profitable.

The social feature of the occasion appears to have been quite prominent. Benefits thus derivable are by no means of minor importance.

The American Dental Convention was organized about sixteen years ago; it grew out of the demands of the profession at that time; the profession advanced, but the Convention did not, in its organic character, at least, sufficiently to answer the requirements of the rapid progress that was being made, and so another body was established. But the Convention did not abandon the field; indeed it has had a work to do, a mission to fulfill, and it is accomplishing it. We hope it will continue as long as it is a means of good to the profession.

It meets again next year, at the same place. Dr. John Allen, of New York, is President, and Dr. C. S. Hurlbut of Springfield, Massachusetts, Secretary.

### ROSE PEARL.

In the August number of the REGISTER, we made some reference to Rose Pearl as a base for artificial dentures; and we have now only to say that from the additional experience of one month, we have no modification to make of what we then said, except that we will add that far longer time is required to test any such thing, than has yet been given to *Rose Pearl.* We will add, also, that the difficulty attaches to this that does to almost all new things, viz: that of efficient manipulation and use from hastily written and imperfect directions. Not one in twenty in the profession can take up any new process or mode and operate it perfectly, without personal instruction; and yet almost any one is willing to make trial from printed description of any new thing, and necessarily failure follows, from the fact that minor details will be overlooked in directions, or not carried out in execution, unless seen; and then comes disgust and abandonment.

### MAD RIVER DENTAL SOCIETY.

The semi-annual meeting of the Mad River Dental Society, will be held at the Phillips House, in Dayton, Tuesday, Oct. 7th, 1873, at 10 o'clock A. M.

ORDER OF EXERCISES:

1 Prayer;
2 Calling the Roll;
3 Reading minutes of last Meeting;
4 Reports of Standing Committees;
5 Reports of Special "
6 Reports of Officers;
7 Election of New Members;
8 Election of Officers;
9 Reading of Essays and Papers;

SUBJECTS FOR DISCUSSION.

What obstacles prevent a more rapid development and progress of the dental profession? And how can they be removed?

What are the most urgent demands of the dental profession in its present position?

What means can be employed for the better preservation of the teeth?

What degree of importance attaches to the teeth, compared to the other organs of the body?

What assurance is the dentist warranted in giving his patient, as to the preservation of his teeth after they are attacked by decay?

What are the modifying circumstances that occasion various results from operations upon the teeth?

How much is the success of filling teeth due to the mere manipulation?

Papers are expected from:—Dr. J. H. Paine; Dr. Geo. Watt; Dr. J. A. Stipp; Dr. Geo. W. Keely; Dr. A. Berry.

GEO. KEELY, *Rres.*, J. A. STIPP, *Sec'y.*

